# Immune complex formation impairs the elimination of solutes from the brain: implications for immunotherapy in Alzheimer’s disease

**DOI:** 10.1186/2051-5960-1-48

**Published:** 2013-08-09

**Authors:** Roxana Octavia Carare, Jessica Liesbeth Teeling, Cheryl A Hawkes, Ursula Püntener, Roy O Weller, James AR Nicoll, Victor Hugh Perry

**Affiliations:** 1 Faculty of Medicine, University of Southampton, Southampton, UK; 2 Centre for Biological Sciences, University of Southampton, Southampton, UK; 3 Clinical Neurosciences, South Lab and Pathology Block, LD66 (Mailpoint 806), Southampton General Hospital, Tremona Road, Southampton, Hampshire SO16 6YD, United Kingdom

**Keywords:** Alzheimer’s disease, Cerebral vasculature, Perivascular drainage, Basement membranes, Immunotherapy

## Abstract

**Background:**

Basement membranes in the walls of cerebral capillaries and arteries form a major lymphatic drainage pathway for fluid and solutes from the brain. Amyloid-β (Aβ) draining from the brain is deposited in such perivascular pathways as cerebral amyloid angiopathy (CAA) in Alzheimer's disease (AD). CAA increases in severity when Aβ is removed from the brain parenchyma by immunotherapy for AD. In this study we investigated the consequences of immune complexes in artery walls upon drainage of solutes similar to soluble Aβ. We tested the hypothesis that, following active immunization with ovalbumin, immune complexes form within the walls of cerebral arteries and impair the perivascular drainage of solutes from the brain. Mice were immunized against ovalbumin and then challenged by intracerebral microinjection of ovalbumin. Perivascular drainage of solutes was quantified following intracerebral microinjection of soluble fluorescent 3kDa dextran into the brain at different time intervals after intracerebral challenge with ovalbumin.

**Results:**

Ovalbumin, IgG and complement C3 co-localized in basement membranes of artery walls 24 hrs after challenge with antigen; this was associated with significantly reduced drainage of dextran in immunized mice.

**Conclusions:**

Perivascular drainage along artery walls returned to normal by 7 days. These results indicate that immune complexes form in association with basement membranes of cerebral arteries and interfere transiently with perivascular drainage of solutes from the brain. Immune complexes formed during immunotherapy for AD may similarly impair perivascular drainage of soluble Aβ and increase severity of CAA.

## Background

Unlike most other organs in the body, the brain has no conventional lymphatic drainage. However, experimental studies using soluble tracers in mice have shown that solutes injected into the interstitial fluid of the brain parenchyma drain along basement membranes in the walls of capillaries and arteries towards regional lymph nodes in the neck [1,2]. Following injection into the mouse striatum, ovalbumin (OVA, 49 kDa), fluorescent dextran 3-10 kDa, and soluble amyloid-β (Aβ) diffuse through the extracellular spaces of the brain and then enter the basement membranes of cerebral capillaries and arteries that act as the lymphatic drainage pathways of the brain [2]. With age and in Alzheimer's disease (AD), insoluble fibrillary Aβ is deposited in the walls of cerebral capillaries and arteries as cerebral amyloid angiopathy (CAA) [3-5]. Initially, Aβ is deposited in intramural vascular basement membranes that form the lymphatic drainage pathways of the brain but eventually Aβ may occupy the whole thickness of the walls of arteries and capillaries [6-8]. Rupture of amyloid laden vessels is associated with CAA related intracerebral haemorrhage [9,10].

Levels of soluble Aβ are also raised in the brain in AD and this correlates with cognitive decline, suggesting that the elimination of soluble Aβ from the brain may be impaired with age and AD [11,12]. Trials of immunotherapy for AD in humans were introduced following successful studies in transgenic mice showing that insoluble Aβ plaques were removed from the brain following immunization with Aβ42 [13,14]. However, despite the clearance of Aβ plaques from patients with AD, active immunization seems to increase, rather than decrease, the amount of arterial CAA in both transgenic mice and humans [15-17]. It appears that Aβ can be solubilised from plaques but it becomes entrapped in the perivascular drainage pathways manifesting as an increase in the severity of CAA [18]. The dynamics of the events resulting in increased CAA following immunotherapy are not clear but it could reflect either an increased flow of soluble Aβ out of the brain in the perivascular drainage pathways, or it may reflect impaired drainage and elimination of soluble Aβ due to the formation of Aβ-anti-Aβ immune complexes. In addition, trials of both active and passive Aβ antibody therapy in patients with AD have encountered side-effects comprising focal abnormalities in cerebral white matter on imaging, suggesting an increasing fluid in the subcortical white matter, again reflecting failure of fluid drainage from the brain [19]. The pathophysiology underlying the side-effects of Aβ immunotherapy are as yet unclear but may be due in part to the effects of immune complexes forming in perivascular drainage pathways.

In previously published studies, we showed *first* that when OVA is injected into the mouse brain, it drains rapidly out of the brain along basement membranes in the walls of cerebral capillaries and arteries [2]. *Secondly* we showed that active immunization with OVA followed by the intracerebral injection of the antigen OVA, resulted in the formation of immune complexes in the brain parenchyma associated with a robust inflammatory response and macrophage activation [20].

In the present study, we *first* tested the hypothesis that immune complexes form in the interstitial fluid drainage pathways the walls of cerebral arteries of OVA-immunized mice following challenge with the antigen. *Second*, we tested the hypothesis that the presence of OVA immune complexes in cerebral artery walls disrupts the perivascular lymphatic drainage of soluble tracers from the brain.

## Methods

### Animals

BALB/c mice were originally obtained from Charles River (Margate, United Kingdom) and bred and maintained in local facilities. Animal experiments obtained approval from the local Committee for Ethics at the University of Southampton and were performed under Home Office licencing. A total number of 75 mice of 6–10 weeks old were used in this study.

### Active immunization

8 week old Balb/c mice were immunized against OVA by intraperitoneal (i.p.) injection of 50 μg OVA (Sigma-Aldrich, Dorset, UK) in the presence of Alum (1:1 ratio, Alum inject, Fisher Thermo, Loughborough, UK). Mice received a booster injection of 100 μg OVA in saline at 2, 4 and 6 weeks and 3 days before the intracerebral injection of OVA.

### Generation of immune complexes

Mice were anaesthetized by intraperitoneal injection of 0.1 ml/5 g body weight Avertin (2,2,2 tribromoethanol in tertiary amyl alcohol). The scalp was shaved and local anaesthetic (Lignocaine 5% from Biorex Laboratories Ltd) was placed in the external auditory meati as mice were positioned in a stereotaxic frame (Kopf Instruments, Tujunga, CA, USA). 10 mg of OVA was freshly diluted in 1 ml of saline, and 1μl injected into the striatum (bregma 1 mm anterior, lateral 1.5 mm, 2.5 mm deep) of OVA-immunized or control non-immunized mice. Injections were performed over a period of 2 minutes through a glass micropipette with an injecting tip of <50 μm (Sigma-Aldrich, Dorset, UK). Tissue was collected at 5 minutes (n=6), 3 h (n=3), 24 h (n=12), or 7 days (n=12) after the injections. For analysis of immune complex formation, mice were perfused with heparinized saline and brains immediately frozen in Tissue Tek OCT (Sakura Finetek Europe B. V., Zoeterwoude, The Netherlands). Blood samples were taken to assess OVA-specific antibody titers by ELISA and described before [20]. The brains were trimmed to isolate coronal blocks of 7 mm thickness that contained the injection site in the centre and cut into 10 μm thick coronal sections on a cryostat. Sections were collected on APES coated slides for histological examination, quantification studies and immunocytochemistry.

Sections of brain were stained by immunohistochemistry to identify elements characteristic of immune complexes and to study their position in relation to cerebral vascular basement membranes. Complement was identified by antibodies against mouse C3, (FITC conjugated rabbit anti-C3, MP Biomedicals, France), at a dilution of 1:2000. Mouse IgG was identified using FITC labelled F(ab’)_2_ fragments of goat anti-mouse IgG (Sigma-Aldrich, Dorset, UK), at 1:500 dilution. Rabbit anti-OVA (Sigma-Aldrich, Dorset, UK) was used at a dilution of 1:1000. Activated macrophages were studied using an F4/80 monoclonal antibody, (AbDserotec, Oxford, UK) at 1:500 dilution.

To assess the distribution of the immune complexes in relation to the cerebrovascular basement membranes, simultaneous staining of the laminin component of vascular basement membranes was performed by immunocytochemistry using a pan-laminin polyclonal rabbit antibody (Sigma-Aldrich, Dorset, UK) at a 1:500 dilution. Smooth muscle cells of the tunica media of arteries were identified by detecting α-smooth muscle actin using a mouse monoclonal antibody (Sigma-Aldrich, Dorset, UK) at 1:4000 dilution.

### Injections of fluorescent tracer

**Figure 1:**
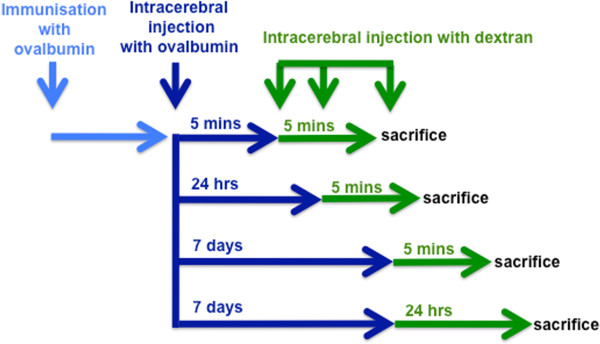
**Schematic demonstrating the temporal sequence of immunization and injection experiments.** Wild type BALB/c mice were immunized with OVA and then injected with OVA in the striatum at different time points. A soluble fixable fluorescent dextran was then injected intracerebrally at 5 mins or 24 h or 7 days post-immunization. Mice were examined at 5 minutes after injections of dextran.

At 5 minutes, 24hr and 7 days following the intracerebral injections of OVA, fluorescein labeled 3 kDa soluble lysine fixable dextran (Invitrogen, Paisley, UK) was injected into the striatum, as described previously [2] (Figure 1). Dextran was injected at 1 μg/μl in a volume of 0.5 μl, over a period of 2 minutes through a glass micropipette with the injecting tip <50 μm (Sigma-Aldrich, Dorset, UK) as previously described [2]. Mice were sacrificed at 5 minutes following injection of the tracer.

Tissue was collected after terminal anaesthesia with sodium pentobarbitone (250–300 μl intraperitoneally) and transcardially perfusion with heparinized 0.9% saline followed by 4% paraformaldehyde in 0.1 M phosphate buffer pH 7.4. Brains were removed and further fixed by immersion in 4% paraformaldehyde for 4–6 hours and placed in 30% sucrose for 48 h for cryoprotection. The brains were trimmed to form coronal blocks 7 mm thick with the injection site in the centre. Blocks were then frozen in Tissue Tek OCT (Sakura Finetek Europe B. V., Zoeterwoude, The Netherlands) and sectioned in a coronal plane (10 μm thick) on a cryostat. Sections were collected on gelatin-coated slides for histological examination, quantification studies and immunocytochemistry.

Secondary antibodies or streptavidin (in the cases where avidin-biotin complex was used) labeled with Alexa Fluor 488 or Alexa Fluor 546 fluorochrome (Invitrogen, Paisley, UK) to enable visualization with a fluorescence microscope. The specificity of the labeling was controlled by omission of the primary antibody. Mounted sections were cover slipped using Vectashield (Vector Labs, Peterborough, UK).

### Quantitation of interstitial fluid drainage

Images of 1030 × 1030 pixels per inch were obtained at ×25 magnification at an SP2 Leica Confocal System using sequential scanning. Co-localization of fluorescent markers appeared yellow. The illumination of the specimen provided by the SP2 confocal laser scanning microscope used in this study was restricted to a single point (~ 0.25 μm in diameter by ~ 0.5 μm deep), scanned across the specimen. By inserting a confocal imaging aperture into the light path, the resulting image comprises mainly in-focus information from the focal plane whilst the majority of the out-of-focus flare (associated with conventional microscopy) was eliminated. Thus, a series of in-focus 1 μm optical sections were acquired and combined to produce a sharp 3 dimensional image of the whole specimen.

#### Co-localization technique

When two spectra for different labels (i.e. double labeling) are acquired, two data sets are produced (one for each fluorochrome). The SP2 Leica software compares the labeling intensity from the two data sets, voxel by voxel; if there is no labeling in either corresponding voxel it appears black, labeling in one but not the other voxel appears green (or red). Whenever a high intensity of labeling in both channels is detected, the programme converts the point to a chosen colour (for images in this thesis, blue or yellow). To minimize false positives due to fluorescence detected in both channels (“bleed through”), sequential rather than simultaneous acquisition of data sets for each fluorochrome was performed.

The *number* of blood vessels with dextran in their walls or in perivascular cells was quantified within the confocal images, using the KS-400 software on a Zeiss Image Analysis. Five images were taken for each section. The area within which the blood vessels were counted in each image was of 391193 μm^2^. The number of blood vessels outlined with dextran was used for graphical and statistical analysis. All statistical tests were carried out using SPSS 16.0. Two way ANOVA test with Bonferroni correction was applied and values of p<0.05 were considered statistically significant. The *type* of blood vessels was determined by their diameter and the presence of smooth muscle actin. Vessels were considered to be capillaries if their diameter was under 10 μm and if they lacked smooth muscle actin staining; arteries were defined by a diameter greater than 10 μm and by the presence of smooth muscle actin staining in their walls. Veins were larger than 10 μm in diameter and lacked smooth muscle actin.

## Results

### Relation of immune complexes to the cerebral vasculature following intracerebral injection of ovalbumin in ovalbumin- immunized mice

The immunization protocol has been shown in previous studies to generate high levels of circulating OVA specific antibodies, with half maximal binding within dilutions of 1:10^5^ -1:10^7^, demonstrated in a previous related study [20].

**Figure 2:**
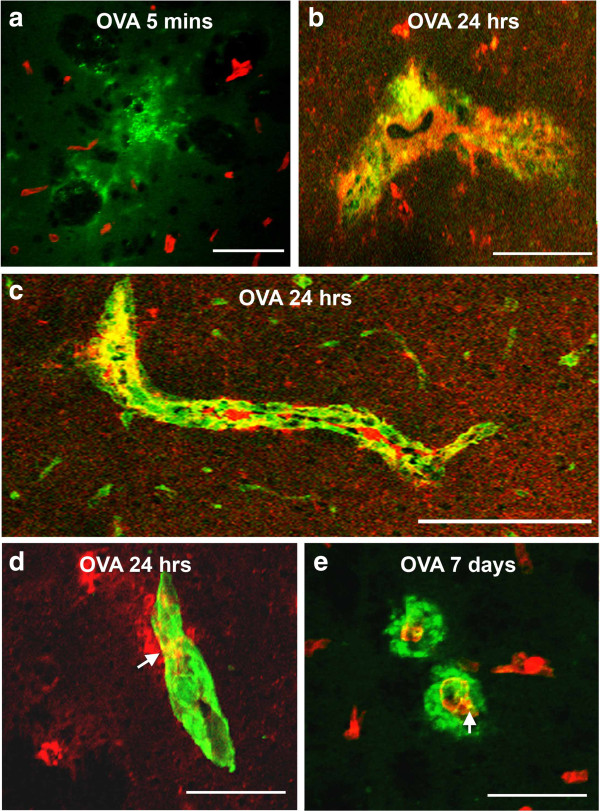
**Immune complexes in basement membranes in the walls of cerebral capillaries and arteries.** Active immunization with OVA was followed by intracerebral challenge with OVA and left in situ for **(a)** 5 mins, **(b-d)** 24 hours and **(e)** 7 days. **(a)** At 5 mins, complement C3 (green) is diffusely in the vicinity of blood vessels stained for laminin (red); there is no co-localization of complement and laminin. **(b)** At 24 hours, IgG (red) is seen distributed diffusely in the perivascular brain parenchyma and is co-localized (yellow) with complement C3 (green) in brain parenchyma. **(c)** At 24 hours after injection of antigen, a longitudinal section of an artery in the striatum shows co-localization (yellow) of complement (green) with laminin (red) in the basement membranes surrounding smooth muscle cells in the tunica media. **(d)** IgG (red) is present in the brain parenchyma and co-localizes (yellow) with laminin (green) in a blood vessel wall at 24 hours. **(e)** At 7 days, complement C3 (green) is distributed circumferentially around blood vessels (red=laminin). Some co-localization of complement C3 and laminin (yellow) is still seen in the blood vessel walls. Confocal images: co-localization appears as a yellow colour. Scale bars = 60 μm.

We previously showed that intracerebral injection of OVA in OVA-immunized mice results in immune complex formation and inflammation around cerebral blood vessels [20]. In the current study we aimed to investigate the kinetics and location of the immune complexes in more detail and to test the hypothesis that the perivascular drainage of solutes is affected under these conditions. To analyze immune complex formation following intracerebral OVA injection at different time points, we first stained tissue from non-immunized and OVA immunized mice for OVA, IgG, and C3. Laminin was used to identify the location of the immune complex within the blood vessel wall (Figure 2). *Five minutes* and *three hours* after its injection in immunized mice, OVA was diffusely spread in the ipsilateral striatum, corpus callosum and cortex as described previously [2]. Around the injection site for OVA, Complement C3 and IgG were present on both sides of the laminin of capillaries of OVA immunized mice (Figure 2a). IgG was seen within the lumen of blood vessels, with little extravasation in the perivascular space. The staining for C3 was diffuse and limited only to the injection site, without any C3 observed in basement membranes. Non-immunized control mice showed minimal levels of IgG and C3 (data not shown).

**Figure 3:**
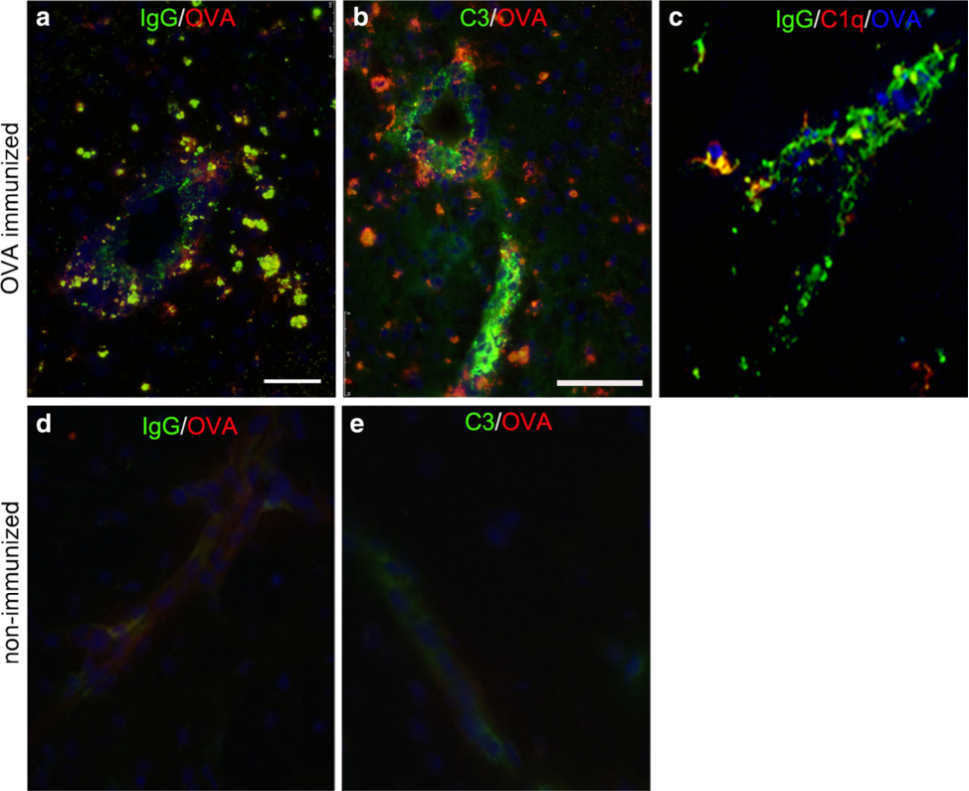
**Immune complexes.** Immune complex formation was analyzed at 24 h after intracerebral OVA injection and stained for: **a** and **d)** IgG(green) and OVA (red), **b** and **e)** C3 (green) and OVA (red) and **c)** IgG (green), C1q (red) and OVA (blue). Co-localization is shown in yellow. Representative of n=3. Scale bar =75 am.

At *twenty*-*four hours*, IgG, C3 and OVA were identified in the brain in close proximity to the cerebral vasculature and double staining for IgG and laminin revealed that IgG and complement C3 were present in the parenchyma and also co-localized with laminin present in the walls of blood vessels (Figure 2b-d). Staining for IgG, OVA, C3 and C1q confirmed the presence of immune complexes in the parenchyma and their association with blood vessels (Figure 3). OVA was no longer detectable in association with the cerebrovascular basement membranes at 7 days (Figure 2e).

### Reduced perivascular drainage of dextran tracer in the presence of immune complexes

Having demonstrated that immune complexes are formed in the basement membrane, 24 h, but not yet at 5 minutes, after intracerebral injection of OVA, we next investigated perivascular drainage in the presence of immune complexes by co- injecting a fluorescent tracer at 5 minutes, 24 h and 7 days following the intracerebral injection of OVA.

We previously showed that within five minutes of its injection, fluorescent dextran spreads diffusely and is found at the basement membranes of cerebral capillaries of the grey matter [2]. Therefore we used this time point to test if drainage of dextran is impeded following immune complex formation in the basement membranes of cerebral blood vessels. In a separate group of mice, we allowed dextran to drain for 24 h.

**Figure 4:**
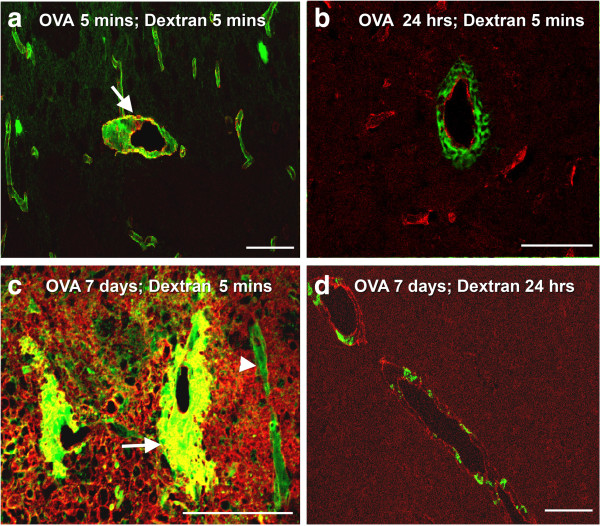
**Perivascular drainage of dextran. (a)** Following active immunization with OVA, at 5 minutes after the intracerebral injection of OVA and 5 minutes after the injection of dextran (green), the distribution of dextran is diffuse in the extracellular spaces and within the walls of capillaries and arteries. The arrow points to an artery with smooth muscle staining (red). **(b)** Following active immunization with OVA, at 24 hours after the intracerebral injection of OVA, the dextran injected intracerebrally (green) is present within 5 minutes of its injection as cuffs around vascular laminin (red). **(c)** In mice actively immunized with OVA, at 7 days after the intracerebral injection of OVA, dextran (green) is in the walls of blood vessels (arrowhead) and present within cuffs around large blood vessels (arrow). **(d)** In mice actively immunized with OVA, at 7 days after the intracerebral injection of OVA and 24 h after the intracerebral injection of dextran (green), the dextran is present in cells within a perivascular location (red-laminin). Confocal images: co-localization appears as a yellow colour. Scale bars: a, b, d = 100 μm; c = 120 μm.

**Figure 5:**
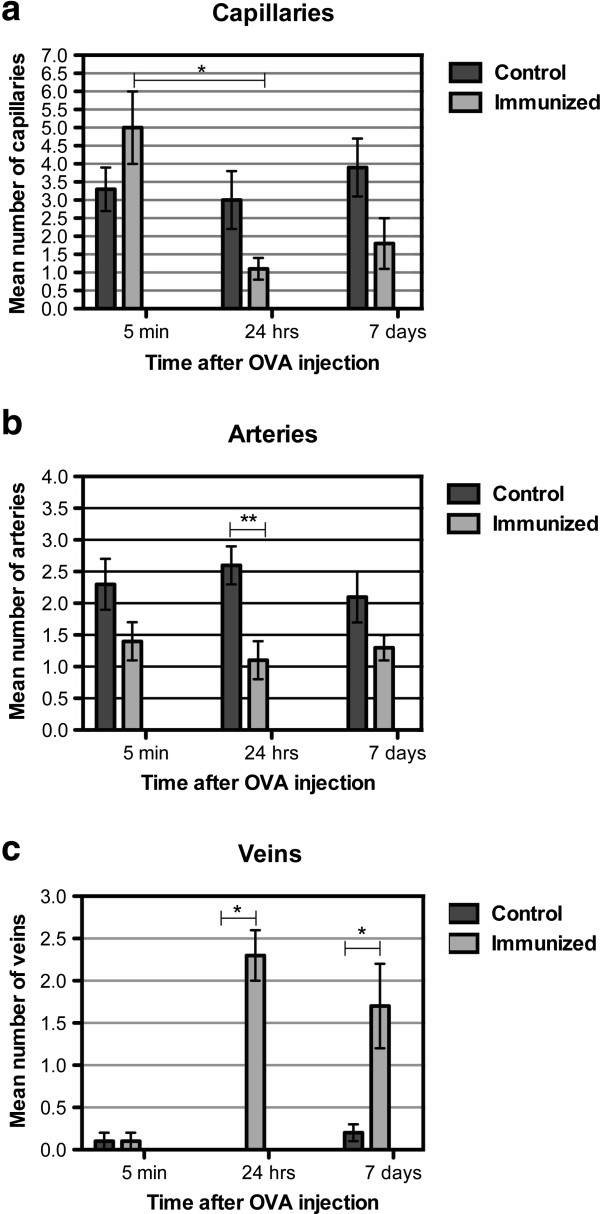
**Quantification of the number of blood vessels labeled with fluorescent dextran following the formation of immune complexes.** Control (non-immunized) and OVA-immunized mice where OVA was left intracerebrally for 5 minutes, 24 h, 7 days, followed by the injection of fluorescent dextran and animals sacrificed within 5 mins. **(a)** The number of capillaries in control and OVA immunized mice, following intracerebral injection of OVA at 5 minutes, 24 h, or 7 days. The number of capillaries labeled with dextran is significantly lower in immunized animals, analyzed 24 h after OVA injection, compared to analysis at 5 minutes. **(b)** The number of arteries in control and OVA immunized mice, following intracerebral injection of OVA at 5 minutes, 24 h, or 7 days. The number of arteries labeled with dextran is significantly lower in OVA immunized mice where immune complexes were left to form for 24 hours, compared to the control non-immunized group. **(c)** There is a significant difference between the number of veins with dextran in a perivascular location in the OVA immunized mice analyzed at 24 h or 7 days, compared to control non-immunized mice. *p≤0.001; **p<0.005.

*Five minutes* after OVA injection, we observed a diffuse distribution of the fluorescent tracer dextran in the extracellular space and within the walls of capillaries and arteries of OVA immunized mice (Figure 4a). This pattern was similar to non-immunized, control animals (data not shown). Quantification of the type of vessels that contained dextran associated with their walls revealed that at 5 minutes after injection of OVA, there was no difference in the number of dextran positive arteries or capillaries, compared to non-immunized controls (Figure 5a and b). Furthermore, there were no differences in the number of veins labeled with dextran between this group and control animals (Figure 5c).

At 24 h after OVA injection, dextran had a reticular cuff appearance around laminin in the walls of capillaries and arteries (Figure 4b). Quantification of the number of blood vessels that contained dextran associated with the basement membranes revealed no difference in the number of capillaries with dextran in their walls, compared to non-immunized control mice (Figure 5a). A statistically significant reduction in the number of arteries with dextran in their walls was observed compared to controls (p=0.003, Figure 5b). We further showed that dextran positive veins are significantly higher (p<0.001, Figure 5c) in OVA-immunized mice when analyzed 24 h after intracerebral OVA injection.

**Figure 6:**
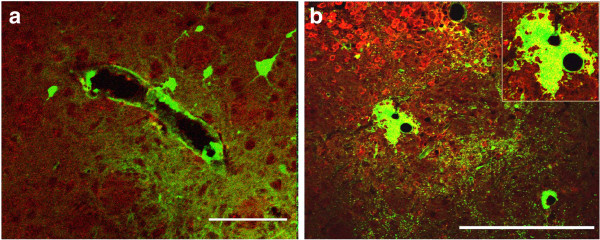
**Active immunization with OVA followed by intracerebral challenge with OVA that was then left in situ for 7 days.** Immune complex formation induces expression of F4/80 on perivascular macrophages and microglia and changes the pattern for the diffusion and elimination of dextran. **(a)** In non-immunized control mice, dextran (green) outlines the contour of a large blood vessel. Immunofluorescence for F4/80 reveals no staining. **(b)** 7 days following immune complex formation, F4/80 staining (red) is seen in the parenchyma and adjacent to blood vessels. Dextran (green) forms a large cuff around a blood vessel and is closely associated with the staining for F4/80. The yellow staining within the dextran cuff (inset) indicates co-localization with F4/80. Scale bars = 100 μm.

At 7 days after immune complex formation the pattern of distribution of dextran was remarkably different between the OVA-immunized and non-immunized groups of mice. In OVA-immunized mice, the diffuse dextran in the extracellular space was confined only to the injection site. The injected dextran outlined the basement membranes of some blood vessels but was also associated with thick perivascular cellular cuffs around other blood vessels (Figure 4c). Staining with smooth muscle actin and subsequent measurement of the diameter of the blood vessels revealed that dextran was present in the walls of capillaries and arteries but the perivascular cuffs with uptake of dextran was present solely around veins and not around arteries or capillaries. In addition, we detected an increased number of F4/80 positive cells in the injection site (Figure 6b). A statistically significant number of veins contained dextran in the surrounding F4/80 positive cells, compared to non-immunized control animals (p=0.001, Figure 5c), while quantification of the number of capillaries and arteries that contained dextran in association with basement membranes revealed no difference (Figure 5a and 5b).

## Discussion

When OVA is injected into the mouse brain, it diffuses through the extracellular spaces of the grey matter and drains out of the brain along basement membranes in the walls of cerebral capillaries and arteries following the lymphatic drainage pathways of the brain parenchyma [2]. Following immunization of mice using OVA as the antigen, injection of OVA into the brain results in the formation of immune complexes in the parenchyma and this stimulates macrophage invasion and/or microglial activation [20]. The aim of the present paper was to test the hypotheses that a) following the injection of OVA into OVA-immunized mice, immune complexes form within the basement membranes in the walls of cerebral capillaries and arteries and that b) such intramural immune complexes disrupt perivascular lymphatic drainage of solutes from the brain. We present evidence that both these hypotheses have been substantiated.

### Location of immune complexes in the walls of cerebral arteries

The presence of the complement components C1q and C3, IgG, and OVA was used in the present study as a marker for immune complex formation [22]. Such immune complexes were detected in the walls of cerebral arteries, co-localizing with laminin in the intramural basement membranes, 24 hours after the injection of OVA into the grey matter of mice immunized against OVA. Co-localization of the components of immune complexes with laminin was identified by confocal microscopy using immunocytochemistry.

### Immune complexes in the walls of cerebral arteries disrupt perivascular drainage of solutes from the brain

Normally, when solutes of different molecular weights like OVA, monomeric soluble Aβ or 3 kDa dextran are injected into the grey matter of the mouse brain, they diffuse rapidly through the interstitial spaces and can be located in the basement membranes in the walls of cerebral capillaries and arteries within 5 min of injection. In this study, we tested the hypothesis that immune complexes within basement membranes would disrupt the perivascular drainage of solutes. Our results showed disruption of perivascular drainage of dextran 24 hours after the injection of OVA into OVA-immunized mice and at the same time that immune complexes were located in vascular basement membranes. The coincidence of immune complexes in arterial basement membranes and disruption of perivascular drainage of dextran is further emphasized by the finding that immune complexes were no longer present in the basement membranes of arteries 7 days after the injection of OVA in immunized mice.

### Effects of inflammation on perivascular drainage

Seven days following the intracerebral injection OVA into OVA-immunized mice, there was extensive perivenous accumulation of inflammatory cells in the brain. The inflammatory reaction resulted in distortion of distribution of the dextran tracer injected into the brain. Dextran accumulated in the enlarged perivenous spaces and was presumably taken up by activated macrophages. Although there was a trend for a reduced number of capillaries and arteries showing dextran in the walls in the 7 day OVA-immunized animals, it did not reach statistical significance. This suggests that the presence of perivenous inflammatory cuffs at this time-point does not have a significant effect on perivascular drainage of solutes along the walls of capillaries and cerebral arteries.

Perivascular macrophages normally take up tracers such as dextran and Aβ that are injected into the brain parenchyma [2,23]. By 24 hours after the injection of dextran into normal mice, dextran is only present in perivascular macrophages around arteries in the ipsilateral hemisphere and no dextran is detected within basement membranes in artery walls. In immunized mice that had formed immune complexes and were examined 24 hours after the injection of dextran, perivascular macrophages had taken up dextran around arteries in the parenchyma in the vicinity of the injection and around veins. This suggests that perivascular macrophages play a key role in clearing solutes from the brain when perivascular drainage is disrupted.

### Implications for Aβ immunotherapy in Alzheimer’s disease

Following Aβ immunization in AD, there is an increase in Aβ42 in capillary and artery walls [18,24,25]. Aβ40 is usually the predominant amyloid in vessel walls and Aβ42 is detected mainly in parenchymal plaques. Increased amounts of Aβ42 in vessel walls following immunization appear to be the result of solubilization of the parenchymal plaques [18,24] allowing more Aβ42 to reach the drainage channels in capillary and artery walls, accompanied by vasogenic oedema [19].

There are no reports of immune complexes formed in the brains of immunized humans against Aβ. However, it is possible that Aβ solubilized from plaques may encounter Aβ- specific antibodies generated in the process of immunotherapy in the walls of cerebral blood vessels. High levels of complement have been reported in transgenic mice that harbour Aβ mutations and that complement co-localizes with the microvascular Aβ [26]. Increased levels of C3 were also found to associate with increased haemorrhages following experimental immunotherapy [27-30], and de-glycosylated antibodies, with reduced complement binding activity, show reduced haemorrhages in an experimental mouse model, suggesting an important role of the effector function of therapeutic antibodies in the development of these side effects [31]. It has been reported that, following immunotherapy with Aβ in mouse models, small amounts of anti-Aβ antibodies enter the brain and form immune complexes that are cleared from the brain into the blood via the neonatal Fc receptors present ubiquitously in the endothelia [32]. In our model, in wild type mice, we observed accumulation of OVA in perivascular macrophages, arguing against the removal of immune complexes via this receptor. A possible explanation may be due to the high antibody titers in our model, leading to higher levels of immune complexes (excess IgG) that saturate efflux mechanisms via FcRn.

The risk of intracerebral haemorrhage may increase if immune complexes are formed in the basement membranes of capillaries and arteries affected by CAA, as the wall is already structurally weak from the deposition of Aβ and is predisposed to aneurysm and rupture [9,21,33]. Recently it has been shown that active immunotherapy using Aβ derivatives and alum in adult but not old Tg2576 mice results in a reduction of Aβ burden, without worsening of CAA or inducing microhaemmorhages [34]. In the light of the findings of our study, if blood vessels are still unaffected by the morphological changes associated with senescence and deposition of Aβ, they may accommodate the drainage of the Aβ solubilized from plaques, without rupturing the vascular wall. This is particularly important in the light of new immunization protocols that are administered early, before AD pathology is advanced [35,36].

## Conclusions

**Figure 7:**
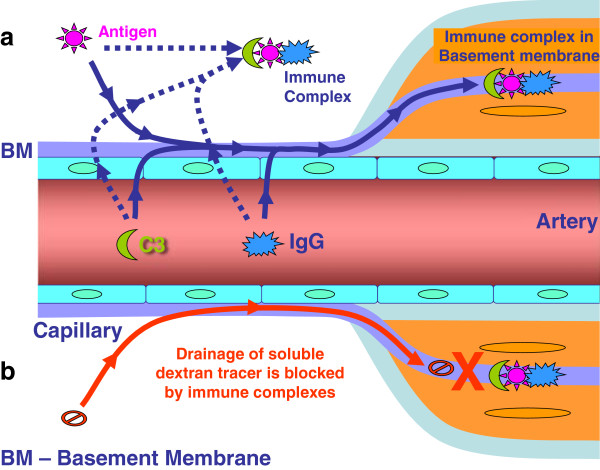
**Diagram to illustrate the possible mechanism by which immune complexes disrupt the perivascular drainage of solutes.** In **(a)**, soluble antigen in the extracellular space interacts with IgG extravasated from the circulation resulting in immune complex formation and fixation of complement C3. The immune complexes formed block the arterial basement membranes that represent the perivascular drainage pathway. In **(b)**, the drainage of a soluble tracer (dextran) is blocked by the presence of immune complexes in the arterial basement membranes. Alternatively, dextran is taken up by activated macrophages and microglia surrounding the blood vessels. We previously showed that macrophages/microglia are activated by FcR ligation following immune complex formation, which initiates an inflammatory in response surrounding the blood vessels (veins). The activated macrophages take up dextran by phagocytosis, thereby indirectly impeding elimination of solutes from the brain.

Our results demonstrate that immune complexes that are present within the walls of arteries interfere with perivascular drainage of solutes. Inflammation associated with the presence of immune complexes results in a sequestering of solutes within inflammatory cells. We do not know whether immune complexes form in the extracellular spaces of the brain and then drain via perivascular routes, or whether they form within the arterial basement membranes (Figure 7). It remains to be clarified to what extent immune complexes are formed after immunization with Aβ and the proportion of capillaries and arteries that remain functional to allow the drainage of solubilized Aβ and other metabolites. Future prophylactic and therapeutic strategies in ageing and CAA will aim to test chaperone molecules that may facilitate drainage along cerebrovascular basement membranes even if immune complexes reside in them.

## Competing interest

The authors declare that they have no conflict of interest.

## Authors’ contributions

RC, VP, ROW, JN and JT designed the study. RC carried out the intracerebral injections of OVA and dextran, prepared the tissue, analyzed the pattern of perivascular drainage drafted the manuscript. JT and UP immunized the mice and analyzed the immune complexes. CH performed the quantification and statistical analysis. All authors contributed to the manuscript, read and approved the final manuscript.
